# OxyR-regulated catalase CatB promotes the virulence in rice via detoxifying hydrogen peroxide in *Xanthomonas oryzae* pv. *oryzae*

**DOI:** 10.1186/s12866-016-0887-0

**Published:** 2016-11-08

**Authors:** Chao Yu, Nu Wang, Maosen Wu, Fang Tian, Huamin Chen, Fenghuan Yang, Xiaochen Yuan, Ching-Hong Yang, Chenyang He

**Affiliations:** 1 State Key Laboratory for Biology of Plant Diseases and Insect Pests, Institute of Plant Protection, Chinese Academy of Agricultural Sciences, Beijing, 100193 China; 2 Department of Biological Sciences, University of Wisconsin-Milwaukee, Milwaukee, WI 53211 USA

**Keywords:** *Xanthomonas oryzae* pv. *oryzae*, Catalase, Hydrogen peroxide, Virulence

## Abstract

**Background:**

To facilitate infection, *Xanthomonas oryzae* pv. *oryzae* (*Xoo*), the bacterial blight pathogen of rice, needs to degrade hydrogen peroxide (H_2_O_2_) generated by the host defense response via a mechanism that is mediated by the transcriptional regulator OxyR. The catalase (CAT) gene *catB* has previously been shown to belong to the OxyR regulon in *Xoo*. However, its expression patterns and function in H_2_O_2_ detoxification and bacterial pathogenicity on rice remain to be elucidated.

**Results:**

The *catB* gene encodes a putative catalase and is highly conserved in the sequenced strains of *Xanthomonas* spp. β-galactosidase analysis and electrophoretic mobility shift assays (EMSA) showed that OxyR positively regulated the transcription of *catB* by directly binding to its promoter region. The quantitative real-time PCR (qRT-PCR) assays revealed that the expression levels of *catB* and *oxyR* were significantly induced by H_2_O_2_. Deletion of *catB* or *oxyR* drastically impaired bacterial viability in the presence of extracellular H_2_O_2_ and reduced CAT activity, demonstrating that CatB and OxyR contribute to H_2_O_2_ detoxification in *Xoo*. In addition, Δ*catB* and Δ*oxyR* displayed shorter bacterial blight lesions and reduced bacterial growth in rice compared to the wild-type stain, indicating that CatB and OxyR play essential roles in the virulence of *Xoo*.

**Conclusions:**

Transcription of *catB* is enhanced by OxyR in response to exogenous H_2_O_2_. CatB functions as an active catalase that is required for the full virulence of *Xoo* in rice.

**Electronic supplementary material:**

The online version of this article (doi:10.1186/s12866-016-0887-0) contains supplementary material, which is available to authorized users.

## Background

Plant innate immune responses to bacterial infection include an oxidative burst through elevating the levels of reactive oxygen species (ROS), which are toxic to bacterial cells and cause damages to proteins, nucleic acids, and cell membranes [1]. Hydrogen peroxide (H_2_O_2_), an important ROS, accumulates and diffuses widely between neighboring xylem elements [2]. Accordingly, pathogenic bacteria need to overcome the stress caused the by H_2_O_2_ and gradually establish their infection in plants [3].

One of the mechanisms of bacterial resistance to is H_2_O_2_ is through antioxidant enzymes including catalases [4]. Bacteria maintain basal oxidative stress resistance, but also possess a highly inducible oxidative stress response that is largely controlled by redox-sensing transcription factors which act as redox-operated genetic switches to activate genes involved in the oxidative stress response [5]. As one of the redox-sensing transcription factors, OxyR has been characterized in a number of pathogenic bacteria [6, 7]. OxyR is a DNA-binding transcription factor that is activated under oxidizing conditions by the formation of a disulfide bond between two cysteine residues [8]. When activated, OxyR regulates the expression of genes involved in detoxification by binding to their promoter regions, thus triggering cellular responses to H_2_O_2_ [5, 9].

Bacterial catalases (CATs) are central components of detoxification pathways, which prevent formation of highly reactive hydroxyl radical by catalyzing the conversion H_2_O_2_ to water and oxygen [10]. Based on their enzymatic properties, catalases fall into three classes including monofunctional heme-containing catalases, bifunctional heme-containing catalase-peroxidases, and nonheme or Mn-containing catalases [11]. Multiple catalase isozymes encoded by different genes have been identified in many bacterial species. Interestingly, these genes have different expression patterns in growth phases and in response to oxidative stress, suggesting that they may have different roles in physiological processes and bacteria-host interactions [12]. Moreover, the transcriptional levels and activities of bacterial catalases were largely induced by H_2_O_2_ [13]. For example, *Xanthomonas campestris* pv. *campestris* and *X. axonopodis* pv. *citri* displayed increased levels of catalases KatE and KatG and H_2_O_2_ resistance after H_2_O_2_ treatment [10, 14, 15]. In addition, several studies have reported that catalase activity was induced during the bacterial infection [3, 16], however, the regulatory mechanism of H_2_O_2_ detoxification and its relation to bacterial virulence remains to be demonstrated.

Bacterial leaf blight caused by *X. oryzae* pv. *oryzae* (*Xoo*) is one of the most devastating diseases of rice, which causes annual yield losses of 10–50 % in many rice growing countries [17–19]. Understanding the molecular mechanisms *Xoo* virulence is pivotal to develop effective disease control strategies [20]. *Xoo* produces several virulence-related factors including exopolysaccharide, extracellular enzymes, toxins, adhesins, and the type III secretion system and its effectors during infection [21, 22]. Furthermore, to facilitate its virulence, *Xoo* might have evolved a mechanism for suppression and evasion of basal defense response of rice, such as employing catalases to detoxify H_2_O_2_ elicited by host innate immunity [23–25]. Detoxification of endogenous H_2_O_2_ that is generated through normal metabolic processes, such as aerobic respiration, is mediated by OxyR via regulating the *ahpC* and *ahpF* genes encoding alkyl hydroperoxide reductase [26, 27]. Deletion of *ahpC* significantly affected H_2_O_2_ accumulation during the rice-*Xoo* interaction [28]. *catB* (*PXO_02830*), *katE* (*PXO_02109*) and *srpA* (*PXO_02864*), three putative catalase genes were revealed in the genome of *Xoo* wildtype strain PXO99^A^ by *in silico* analysis [29]. The transcription of these genes were strongly induced during the bacterial interaction with rice suspension-cultured cells [30]. In addition, in-frame deletion of the *katE* gene significantly attenuated bacterial pathogenesis in rice but not H_2_O_2_ resistance [31]. The regulatory mechanisms and involvement in H_2_O_2_ detoxification and pathogenesis of CatB remain unknown.

In this study, we characterized the regulatory mechanism and function of CatB in H_2_O_2_ resistance and its contribution to virulence in rice. Promoter activities and qRT-PCR assays demonstrated that *catB* gene was transcriptionally responsive to H_2_O_2_ and positively regulated by OxyR. Gene deletion and complementation analysis revealed that CatB greatly contributed to both H_2_O_2_ detoxification and full virulence. This study demonstrates that CatB is one of the key virulence factors to facilitate pathogenesis of *Xoo* in rice via H_2_O_2_ detoxification.

## Results

### Identification, deletion and complementation of the *catB* gene

**Fig. 1:**
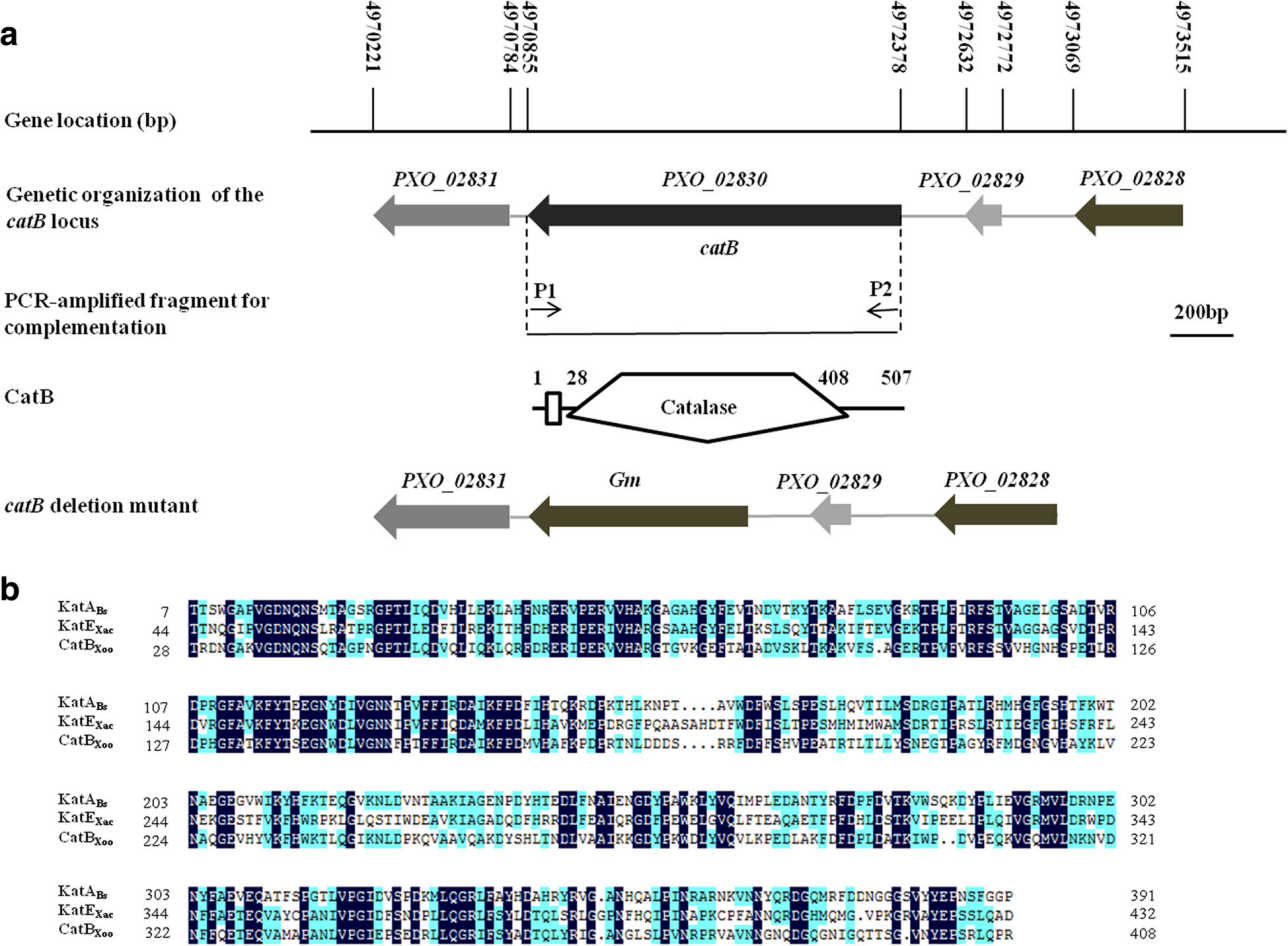
Bioinformatics analysis of *catB* gene. **a** Schematic diagram of the *catB* gene in the genome of PXO99^A^. The open arrows indicate length, location and orientation of the ORFs. The middle element shows *catB* was amplified by PCR primers P1 and P2 and cloned into the plasmid pBBR1MCS-4 for complementation of ∆*catB*. The lower element shows domain structure analyses of the putative CatB protein. The lowest element shows *catB* was exchanged with *Gm* ^*r*^ gene in ∆*catB*. **b** Sequence alignment of CatB (PXO_02830) in *Xoo* PXO99^A^ was performed by using DNAMAN software. KatA and KatE were two catalases which have been experimentally validated in *Bacillus subtilis* (*Bs*) and *Xanthomonas axonopodis* pv. *citri* (*Xac*), respectively. The amino acid residues highlighted with black means the homology level is 100 %, and with blue means the homology level ≥ 50 %

It has been reported that *catB* is one of three putative catalases-encoding genes (*catB* (*PXO_02830*), *katE* (*PXO_02109*), and *srpA* (*PXO_02864*)) that are responsible for H_2_O_2_ degradation in the genome of *Xoo* wildtype strain PXO99^A^ [29]. The open reading frame (ORF) of *catB* is 1,524 bp in length and located in the genome at nucleotide position 4970855–4972378 (Fig. 1a). CatB contains one catalase domain (residues 28–408) with a predicted isoelectric point of 8.66 and molecular weight of 56.4 kD. CatB is also well conserved in other sequenced strains of *Xoo* (MAFF311018, KACC10331, and PXO86) (Additional file 1: Table S1). Additionally, CatB shared over 90 % amino acid identity with those in other important plant-pathogenic *Xanthomonas* species, including *X. campestris* pv. *campestris*, *X. campestris* pv. *vesicatoria*, *X. axonopodis*, *X. perforans*, *X. fragariae*, *X. arboricola*, *X. hortorum*, and *X. fuscans* (Additional file 1: Table S1). Sequence alignment analysis with experimentally validated active CATs, such as KatE from *X. axonopodis* pv. *citri* and KatA from *Bacillus subtilis* revealed that many critical residues in the catalase domain of CatB were probably conserved (Fig. 1b). These observations indicate that CatB might function as an active CAT.

To investigate the potential biological function of CatB in *Xoo*, a *catB* gene deletion mutant (∆*catB*) and complementary strain ∆*catB*(pBBR-*catB*) were constructed as described in the Methods. DNA sequencing analysis showed that the corresponding region of *catB* was replaced by the *Gm* ^*r*^ gene (855 bp in length) in the ∆*catB* mutant. The growth for PXO99^A^, ∆*catB* and ∆*catB*(pBBR-*catB*) were detected in M210 liquid medium. No significant differences were observed in growth rates and bacterial populations between the three strains (Additional file 2: Figure S1). In addition, our previous studies have shown that there was no difference between ∆*oxyR* and wild type in growth in vitro [26]. These results suggest that deletion of *catB* or *oxyR* does not affect the viability of *Xoo* under normal growth conditions.

### *catB* is transcriptionally regulated by OxyR and responsive to exogenous H_2_O_2_

**Fig. 2:**
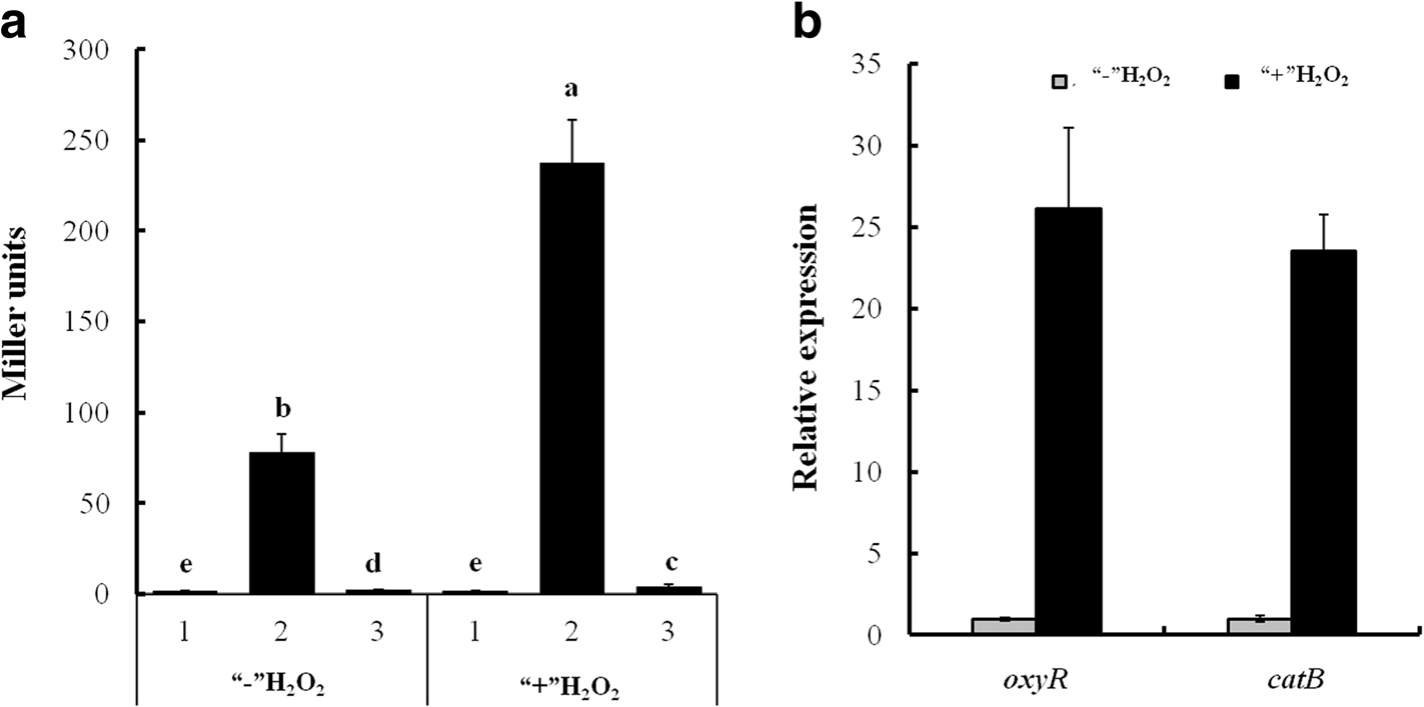
Analysis of *catB* and *oxyR* transcripts in *Xoo* strains. **a** Assays for promoter activities of *catB* in PXO99^A^ and ∆*oxyR* in the presence (“+”) or absence (“-”) of H_2_O_2_. Overnight cultures of wildtype and ∆*oxyR* containing a pH*-catBp-lacZ* transcriptional reporter were inoculated 1:100 into fresh M210 liquid medium and shaken at 28 °C until cells reached at OD_600_ of 1.0, and treated with 3 mM H_2_O_2_ for 0.5 h. The *catB* promoter activity was analyzed by measuring β-galactosidase levels. 1, WT (pH*-lacZ*); 2, WT (pH-*catBp-lacZ*); 3, ∆*oxyR* (pH-*catBp-lacZ*). pH*-lacZ* was an empty plasmid used as the control. **b** Assays for *catB* and *oxyR* transcripts in PXO99^A^ treated with H_2_O_2_. Wildtype cells cultured in M210 liquid medium were exposed to H_2_O_2_ at 3 mM for 0.5 h, H_2_O_2_-untreated cells were used as the control (CK), and the total RNA was extracted with TRIzol reagent. The expression levels of *catB* and *oxyR* were detected by quantitative RT-PCR and normalized to *gyrB*. Bars represent standard errors of the means from three independent cultivations, and different letters above the bars denote statistically significant differences (*P* < 0.05, Student’s *t* test)

**Fig. 3:**
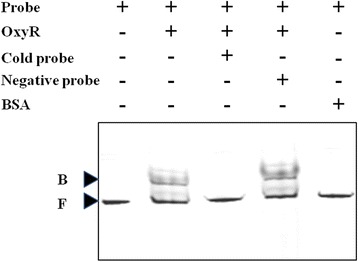
EMSA for OxyR’s binding to *catB* promoter region. Purified OxyR at 5 nM was incubated with 2 nM probe (FAM-labeled *catB* promoter DNA region (length-312/+78)) at 25 °C for 30 min, and the products were run a native 4 % (W/V) polyacrylamide gel in 0.5 × TBE buffer for about 1.5 h at 100 V. Cold probe (unlabeled *catB* promoter DNA region) at 20 nM was used as specific DNA competitor and negative probe (unlabeled coding region of 16S rRNA gene) at 20 nM is used as nonspecific DNA competitor. Bovine Serum Albumin (BSA) at 5 nM was used as the non-specific protein competitor. The addition of OxyR, probes and BSA was indicated by the ‘+’ sign and the omission was indicated by the ‘-’ sign. B: binding probe, F: free probe

OxyR has been shown to function as a transcriptional regulator mediating H_2_O_2_ detoxification in *Xoo*. Our previous studies showed that the *catB* transcripts were significantly reduced in the *oxyR* gene deletion mutant (∆*oxyR*) [26, 30], implying expression of *catB* is regulated by OxyR. To examine whether *catB* is a direct target of OxyR, we first expressed the recombinant OxyR protein in *E. coli* BL21 strain and obtained purified protein (Additional file 3: Figure S2). We then examined the promoter activity of *catB* by the measuring β-galactosidase activities of *catBp*-*lacZ* fusion in various strains. Our results showed that the β-galactosidase activity was 17-fold higher in PXO99^A^ than in ∆*oxyR* (Fig. 2a). Then, electrophoretic mobility shift assay (EMSA) was performed to detect the binding between OxyR protein and the *catB* promoter. The results indicated that the OxyR protein bound directly to the *catB* promoter region (Fig. 3). In contrast, the negative control BSA did not bind to the *catB* promoter region. Addition of unlabeled *catB* promoter DNA fragments as a competitive probes resulted in reduced binding of OxyR with the labeled DNA fragment, while mixture with the 16S rDNA as a non-specific probe did not affected the binding (Fig. 3). These data demonstrated that OxyR regulated the transcription of *catB* gene by directly binding to its promoter region.

To investigate whether their expressions respond to H_2_O_2_ stress, the transcripts of both *catB* and *oxyR* in wildtype PXO99^A^ in the presence or absence of exogenous H_2_O_2_ were assayed by qRT-PCR. The transcription levels of *catB* and *oxyR* were significantly elevated when exogenous H_2_O_2_ was applied (Fig. 2b). In addition, the activity of *catB* promoter in wildtype PXO99^A^ and ∆*oxyR* were measured under exogenous H_2_O_2_ conditions. As expected, the activity of *catB* promoter was dramatically induced in wildtype PXO99^A^ but only slightly increased in ∆*oxyR* in the presence of exogenous H_2_O_2_ (Fig. 2a). Therefore, these results demonstrated that OxyR sensed the presence of H_2_O_2_ and then activated the transcription of *catB.*

### *CatB* and *OxyR* enhance bacterial viability and CAT activity under H_2_O_2_ stress

**Fig. 4:**
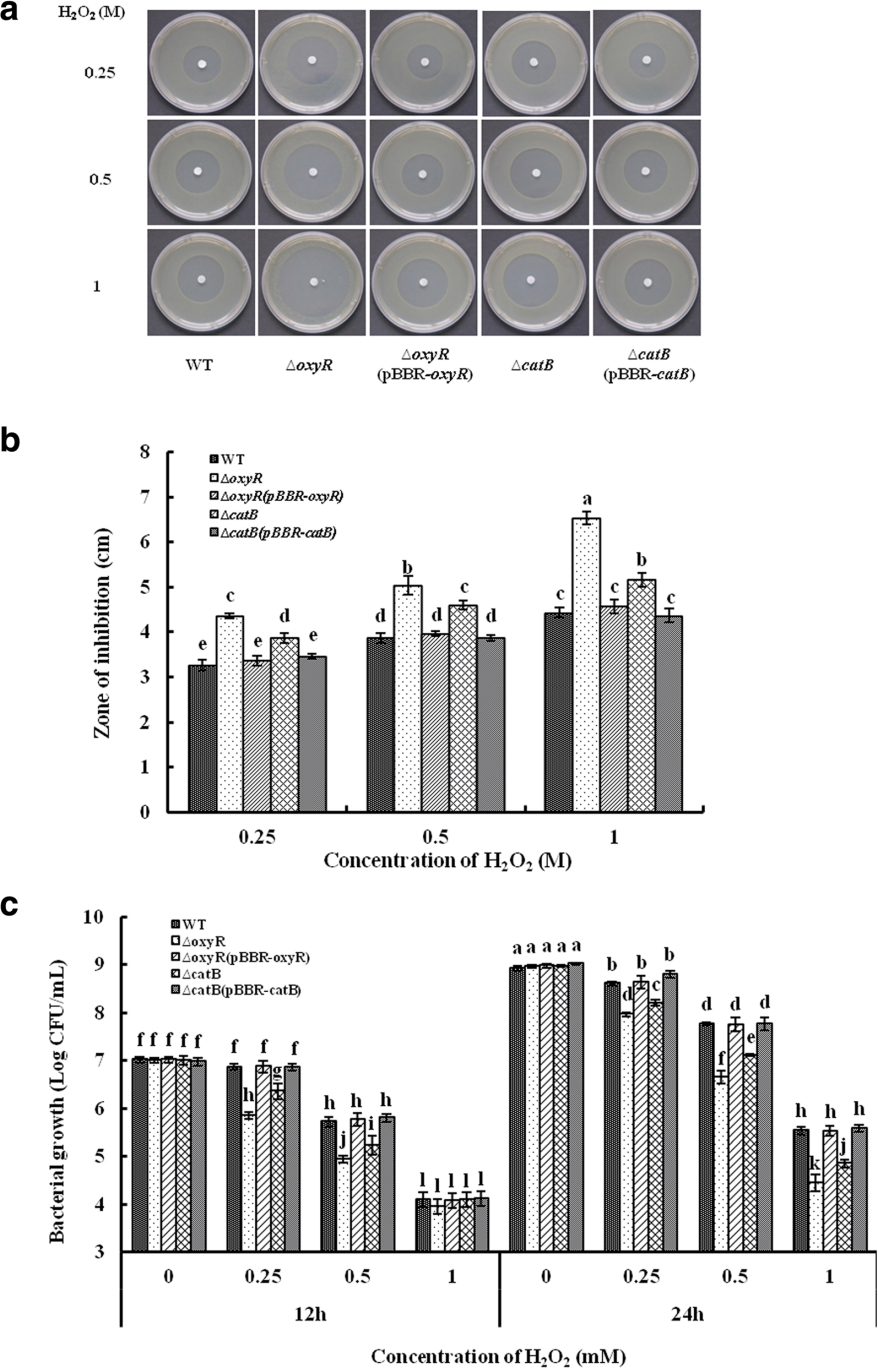
Assays for H_2_O_2_ resistance of *Xanthomonas oryzae* pv. *oryzae* strains. **a** Disk diffusion assays. Wildtype, ∆*catB*, ∆*oxyR*, ∆*catB*(pBBR-*catB*) and ∆*oxyR*(pBBR-*oxyR*) strains at OD_600_ of 1.0 were mixed with PSA medium at 1 : 100 v/v and disks saturated with different concentrations (0.25, 0.5 and 1 M) of H_2_O_2_ were placed on the central of plates. These plates were incubated at 28 °C for 72 h and the H_2_O_2_ inhibition zones were observed. **b** Diameters of the H_2_O_2_ inhibition zones. **c** H_2_O_2_ sensitivity assays. Wildtype, ∆*catB,* ∆*oxyR,* ∆*catB*(pBBR-*catB*) and ∆*oxyR*(pBBR-*oxyR*) strains at OD_600_ of 1.0 were mixed with fresh M210 liquid medium at 1: 1000 v/v, and the H_2_O_2_ were added to the final concentration at 0, 0.25, 0.5 and 1 mM, respectively. The mixtures were incubated at 28 °C with 200 rpm, and the bacterial population were detected at 12 and 24 h. These experiments repeat three times, independently. Error bars represent standard derivations, and different letters above the bars denote statistically significant differences (*P* < 0.05, Student’s *t* test)

To determine the role of *catB* and *oxyR* in H_2_O_2_ resistance, the halo assays of wildtype, ∆*catB*, ∆*oxyR* and their complementary strains ∆*catB* (pBBR-*catB*) and ∆*oxyR* (pBBR-*oxyR*) were performed with the presence of 0.25, 0.5 and 1 M of H_2_O_2_, respectively_._ The sensitivity of bacteria to H_2_O_2_ was indicated by the zone of inhibition. As shown in Fig. 4a and b, the diameters of inhibitory zone for ∆*catB* and ∆*oxyR* were significantly bigger than that of the wildtype at each concentration of H_2_O_2_, while no differences were observed between wildtype and the complementary strains. To further test the H_2_O_2_ sensitivity, the growth ability of PXO99^A^, ∆*catB* and ∆*oxyR* and their complementary strains ∆*catB* (pBBR-*catB*) and ∆*oxyR* (pBBR-*oxyR*) in M210 with the presence of 0, 0.25, 0.5 and 1 mM of H_2_O_2_, respectively, were detected. In the absence of H_2_O_2_, there was no significant difference in growth rate and bacterial population at 12 and 24 h among these strains (Fig. 4c). Compared with the wild type, ∆*catB* and ∆*oxyR* showed more sensitivities at the concentration of 0.25 and 0.5 mM of H_2_O_2_ in M210, and their complementary strains were restored to the wild-type levels (Fig. 4c). Moreover, the growth rates of these strains were significantly inhibited and the bacterial numbers were dramatically decreased in the presence of 1 mM of H_2_O_2_ (Fig. 4c). This demonstrates the essential role of CatB and OxyR in protecting bacterial viability under H_2_O_2_ stress in *Xoo*.

**Fig. 5:**
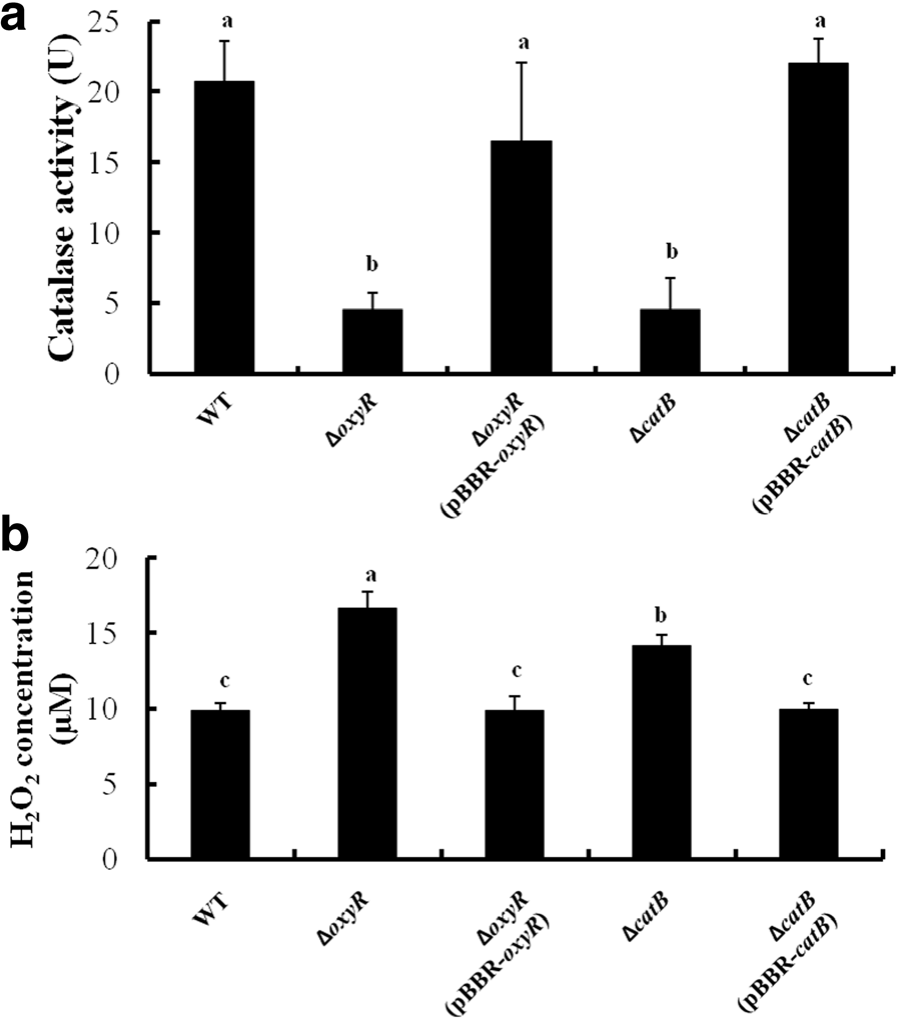
Assays for catalase activity and H_2_O_2_ accumulation of *Xanthomonas oryzae* pv. *oryzae* strains. **a** Catalase activity assays. The cells of wildtype, ∆*catB*, ∆*oxyR*, ∆*catB*(pBBR-*catB*) and ∆*oxyR*(pBBR-*oxyR*) were disrupted by sonication, and the cell extracts were separated by centrifugation at 12,000 g. Catalase activities associated with cell extracts were assessed by spectrophotometric assay. One unit (U) is defined as the amount of activity required to decompose 1 μmol of H_2_O_2_ in one minute. **b** H_2_O_2_ accumulation assays. The H_2_O_2_ concentrations of *Xoo* strains were measured as described in Methods, and the account of H_2_O_2_ was accounted by the standard curve. Error bars represent standard derivations from three replicates, and different letters above the bars denote statistically significant differences (*P* < 0.05, Student’s *t* test)

To further unveil the function of *catB* and *oxyR* in H_2_O_2_ degradation, we comparatively measured the CAT activities of PXO99^A^, ∆*catB*, ∆*oxyR*, ∆*catB* (pBBR-*catB*) and ∆*oxyR* (pBBR-*oxyR*). The catalase activities of ∆*catB* and ∆*oxyR* were significantly decreased compared to that of PXO99^A^, but were restored in their complementary strains ∆*catB* (pBBR-*catB*) and ∆*oxyR* (pBBR-*oxyR*) (Fig. 5a). In addition, the H_2_O_2_ accumulations of these strains were measured. Compared with wildtype, the H_2_O_2_ concentrations were significantly increased in ∆*catB* and ∆*oxyR*, but were restored in their complementary strains (Fig. 5b). Our results suggested that CatB and OxyR contributed to CAT activity for H_2_O_2_ detoxification in *Xoo*.

### *ΔcatB* and *ΔoxyR* elicit early H_2_O_2_ production in rice

In response to pathogenic infection, host plants produce ROS to defend themselves [32]. To evaluate their abilities in the induction of ROS, H_2_O_2_ production were detected in rice leaves at 12 and 24 h post-inoculation of wildtype, ∆*catB*, ∆*oxyR*, ∆*catB*(pBBR-*catB*), and ∆*oxyR*(pBBR-*oxyR*) strains by using 3, 3′-diaminobenzidine (DAB) staining. Similar dark spots formed by DAB in the presence of H_2_O_2_ were observed in all bacterium-inoculated areas at 12 h, whereas no H_2_O_2_ accumulation was found in the H_2_O control (Additional file 4: Figure S3). The same results were observed for all treatments at 24 h post-inoculation. Thus, these findings indicate that wildtype, ∆*catB* and ∆*oxyR* induce H_2_O_2_ accumulation in rice at early stage of infection.

### *ΔcatB* and *ΔoxyR* showed attenuated virulence and bacterial growth in rice

**Fig. 6:**
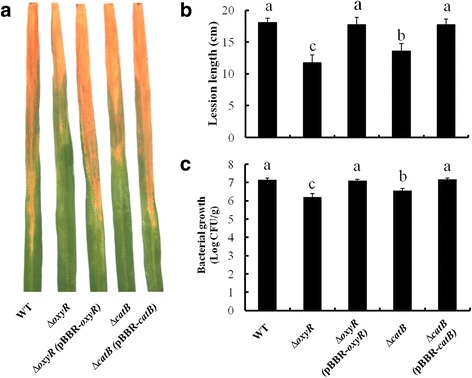
Virulence test of *Xanthomonas oryzae* pv. *oryzae* strains in rice. **a** Wildtype, ∆*catB*, ∆*oxyR*, ∆*catB*(pBBR-*catB*) and ∆*oxyR*(pBBR-*oxyR*) strains were inoculated on the rice leaves (6 weeks old) by using the leaf-clipping method. The disease symptoms were observed at 14 days post-inoculation. **b** The lesion lengths were recorded from 10 inoculated leaves for every strain. **c** Bacterial numbers in the top 20 cm of each lesion leaf were scored. Data represent the mean and standard deviations of three independent experiments, and different letters above the bars denote statistically significant differences (*P* < 0.05, Student’s *t* test)

To determine the functions of CatB and OxyR in virulence, pathogenicity tests for wildtype, ∆*catB*, ∆*oxyR*, ∆*catB* (pBBR-*catB*) and ∆*oxyR* (pBBR-*oxyR*) on susceptible rice plants were performed. The bacterial cells were inoculated onto the tip of rice leaves by leaf-clipping. The bacterial blight symptoms of rice were scored 14 days after bacterial inoculation. Compared with PXO99^A^, ∆*catB* and ∆*oxyR* displayed reduced disease severity with shorter lesion lengths (Fig. 6a and b), and decreased bacterial growth in rice leave tissues, while ∆*catB*(pBBR-*catB*) and ∆*oxyR*(pBBR-*oxyR*) showed disease phenotypes at near-wildtype levels (Fig. 6c). These observations demonstrated that the CatB and OxyR were required for the full virulence and *in planta* growth of *Xoo* in rice.

## Discussion

Pathogenic bacteria successfully survive in the environment and infect plant tissues in part by depending on their abilities to counteract oxidative stresses including H_2_O_2_, which can penetrate through bacterial membranes to affect a variety of cellular processes [1]. Since *Xoo* is constantly exposed to H_2_O_2_, its catalases are very likely to be critical to the H_2_O_2_ detoxification process, whether it is produced endogenously through normal aerobic respiration, or the oxidative burst of rice plant cells during plant-pathogen interactions. In the current study, we revealed that CatB, working with OxyR, played a crucial role in H_2_O_2_ resistance and bacterial virulence in *Xoo* by using bioinformatics and genetic analysis approaches. To our immediate knowledge, CatB is the first catalase functionally characterized to facilitate pathogenesis via H_2_O_2_ detoxification in *Xoo*.

Bacterial catalases have been reported to be involved in the H_2_O_2_-degradation pathway and increased tolerance to oxidative stress [13]. *Xoo* depends on a diverse repertoire of antioxidative enzymes to detoxify H_2_O_2_ for its in vitro growth and survival under different H_2_O_2_ stress conditions [26]. Transcription of three catalase genes *catB*, *katE* and *srpA* were strongly induced by exogenous H_2_O_2_ and during the bacterial interaction with rice suspension-cultured cells in *Xoo* [30]. Gene deletion of *katE* remarkably reduced bacterial growth in vitro and diseases leaf lesion of rice, but did not attenuate CAT activity and bacterial resistance to exogenous H_2_O_2_ [31]. The physiological role of SrpA in *Xoo* needs to be studied in the future. In this study, we identified a catalase gene encoded CatB in *Xoo*, which shared high homology with two validated catalases KatE and KatA from *X. axonopodis* pv. *citri* and *Bacillus subtilis,* respectively (Fig. 1). Deletion of *catB* dramatically attenuated exogenous H_2_O_2_ resistance and CAT activity (Figs. 4 and 5), but did not affect the bacterial growth in vitro (Additional file 2: Figure S1). These results suggested that CatB was one of the key players participating in the H_2_O_2_ degradation pathway in *Xoo*. Furthermore, the homologues of CatB were widely found in other important plant-pathogenic *Xanthomonas* species and pathovars (Additional file 1: Table S1), suggesting that there might be a conserved and functional H_2_O_2_ resistance mechanism by CatB and its homologs in *Xanthomonas* species.

OxyR was identified as the primary H_2_O_2_ sensor responsible for H_2_O_2_ resistance in gram-negative bacteria [8, 33]. Previous studies showed that the cysteine residue (C199) of OxyR in *E. coli* was directly oxidized by H_2_O_2_ to a sulfenic acid, and generated a disulfide bond [34]. This oxidation process activated OxyR as a transcription factor. In *Pseudomonas aeruginosa*, the expression of OxyR was dramatically increased by H_2_O_2_, and mutation of three cysteine sites (C199S, C208S and C296S) for OxyR displayed hypersensitivity to H_2_O_2_ [35]. In this study, the transcription level of *oxyR* was significantly induced by exogenouse H_2_O_2_ (Fig. 2b), implying that OxyR might have similar function to sense H_2_O_2_, thereby activating the downstream target gene expression in *Xoo*.

As a transcriptional activator in response to H_2_O_2_, OxyR has the ability to directly regulate the transcription of target genes through binding to the upstream DNA region of their promoter [36]. The binding sites of four OxyR regulated genes, *katA*, *dps*, *ftn* and *cydA*, were studied in *Corynebacterium glutamicum* [37]. A 50-bp region protected by the OxyR protein in the promoter of each gene was identified using DNase I footprinting analyses. However, no significant sequence similarity was found among the four OxyR-binding sites [37], suggesting the binding sites may vary in different promoters. In this study, we demonstrated that OxyR directly bound to the DNA fragment between −312 and + 78 bp with respect to the translation start site of *catB* in *Xoo* using EMSA (Fig. 3). In addition, the promoter activity of *catB* was significantly reduced in *oxyR* deletion mutant (Fig. 2a). These results suggested that OxyR positively regulated the transcription of *catB* by binding to its promoter region. The OxyR binding sites in the *catB* promoter region will be further studied in the future.

OxyR plays a role during the peroxide stress response by regulating the transcription of catalase genes has been reported in various bacteria, but the regulatory mechanisms are different [5, 16]. For example, OxyR functioned as a positive regulator to activate the expression of catalase genes, and the *oxyR* deletion mutant was hypersensitive to hydrogen peroxide in *Escherichia coli* and *Salmonella typhimurium* [38]. In contrast, OxyR in *Corynebacterium diphtheria* repressed catalase production, and the *oxyR* deletion mutant displayed increased tolerance to H_2_O_2_ [39]. In this study, the promoter activity of *catB* in wildtype *Xoo* strain was significantly enhanced by H_2_O_2_, but dramatically decreased in ∆*oxyR* (Fig. 2a). In addition, deletion of *oxyR* or *catB* significantly reduced the tolerance to H_2_O_2_ and catalase activity (Figs. 4 and 5). These results showed that OxyR acts as a positive regulator to mediate H_2_O_2_ detoxification via controlling *catB* gene transcription in *Xoo*.

Our earlier studies have revealed that OxyR mediated endogenous H_2_O_2_-degradation by regulating the alkyl hydroperoxide reductase genes *ahpC/ahpF* in *Xoo* [26, 27]. Moreover, the transcriptional levels of catalase gene, *katE* and *srpA*, were down-regulated in ∆*oxyR* [26]. These results showed that several genes related with H_2_O_2_ detoxification were regulated by OxyR in *Xoo*. In this study, we showed that ∆*oxyR*, in comparison with ∆*catB*, was more hypersensitive to H_2_O_2_ (Fig. 4), and contained higher level of endogenous H_2_O_2_ (Fig. 5b), suggesting that there might be other genes besides *catB* that are regulated by OxyR in response to H_2_O_2_ in *Xoo*. Interestingly, no significant difference in catalase activity in ∆*catB* and ∆*oxyR* was found (Fig. 5a), implying that the CatB might be one of the major catalases in *Xoo*.

As an important host innate immune response, H_2_O_2_ is generated at the attempted invasion site in plant cells during interactions with potential pathogens and increased host disease resistance [2]. Previoulsy, we have shown that H_2_O_2_ accumulation was induced in rice by *Xoo* infection. Deletion of an alkyl hydroperoxide reductase gene *ahpC* in *Xoo* significantly decreased the endogenous H_2_O_2_ accumulation. However, the H_2_O_2_ scavenging activity was increased with a unknown compensatory mechanism, which led to a lower level of H_2_O_2_ accumulation in the *ahpC* mutant than the wild type during their interactions with rice host plants [28]. These results indicated that AhpC plays the role in the detoxification of endogenous H_2_O_2_ in *Xoo*. In this study, compared with wildtype, ∆*catB* and ∆*oxyR* showed more hypersensitivity to exogenous H_2_O_2_ (Fig. 4) and higher concentration of endogenous H_2_O_2_ (Fig. 5a)_,_ indicating that CatB and OxyR in *Xoo* might be responsible for detoxification of both endogenous and exogenous H_2_O_2._ In addition, H_2_O_2_ accumulations were also observed in rice plants infected by *Xoo* strains (Additional file 4: Figure S3), suggesting that the ability of *Xoo* strains to induce and/or degrade H_2_O_2_ is a key determinant of outcome in the *Xoo*-rice interaction. To further study the differences in H_2_O_2_ accumulation induced by wildtype, ∆*catB* and ∆*oxyR*, quantitative analysis of H_2_O_2_ production in rice and the ability of H_2_O_2_ detoxification in *Xoo* strains would be required.

During evolution, catalase activity has become inducible to help pathogen colonize its host and cause disease by detoxifying H_2_O_2_, implicating that there is a close relationship between bacterial ability to survive H_2_O_2_ stress and its virulence [3, 16]. In *X. campestris*, a catalase KatG is required for virulence in a host plant by providing protection against low levels of H_2_O_2_ [14]_._ Here, we showed the disease severity and bacterial population were dramatically reduced in rice leaves inoculated with ∆*catB* and ∆*oxyR* (Fig. 6), suggesting both ∆*catB* and ∆*oxyR* were significantly attenuated in detoxification of H_2_O_2_ in rice leaves, thereby resulting in the reduced pathogenicity. Based on these observations, combined with our earlier result that *catB* and *oxyR* were transcriptionally induced by H_2_O_2_ produced during interaction with rice suspension-cultured cells and also in real time of infection of rice [26, 30], we propose that both CatB and OxyR are required for full virulence and *in planta* growth of *Xoo* in rice by detoxification of H_2_O_2_. Moreover, shorter lesion lengths and fewer bacterial numbers in rice caused by ∆*oxyR* were observed than that by ∆*catB* (Fig. 6), indicating that there are other virulence factors regulated by OxyR in *Xoo*-rice interactions. This result is consistent with the previous studies that the OxyR regulon comprised of multiple genes involved in H_2_O_2_ detoxification, heme biosynthesis, reductant supply, thiol-disulfide isomerization, Fe-S center repair, iron binding and so on [5]. Accordingly, our identification of CatB regulated by OxyR in *Xoo* highlights the requirement of a functional H_2_O_2_-detoxification for bacterial pathogenesis in rice.

## Conclusions

The *Xoo catB* gene is transcriptionally up-regulated by OxyR in response to H_2_O_2_ either exogenously-applied or generated in rice upon bacterial infection. CatB functions as an active catalase to detoxify H_2_O_2_ and is also required for the full virulence. Thus, OxyR-regulated catalase CatB promotes the bacterial pathogenesis in rice through H_2_O_2_ detoxification.

## Methods

### Bacterial strains and growth conditions

The bacterial strains and plasmids used in this study are listed in Table 1. *E. coli* strains were grown in Luria-Bertani medium at 37 °C. *Xoo* wildtype strain PXO99^A^ and derived mutants were cultured at 28 °C on peptone sucrose agar (PSA) [40] medium or M210 [41] liquid medium with appropriate antibiotics. The antibiotics used were ampicillin (Ap), kanamycin (Km), spectinomycin (Sp) and gentamycin (Gm) at concentrations of 100, 50, 50, and 50 μg mL^−1^, respectively.

**Table 1:** Bacterial strains and plasmids used in this study

Strain or plasmid	Relevant characteristics^a^	Source or Reference
*Escherichia coli*		
DH5α	supE44 ΔlacU169(Φ80lacZΔM15) hsdR17 recA1 endA1 gyrA96 thi-1 relA1	Hanahan 1983 [47]
BL21	For protein expression	Novagen
*Xanthomonas oryzae* pv. *oryzae*		
PXO99^A^	Wildtype strain, Philippine race 6	Lab collection
∆*catB*	*catB* gene deletion mutant derived from PXO99^A^, Gm^r^	This study
∆*catB(*pBBR*-catB)*	Complementary bacterium strain of ∆*catB,* Ap^r^	This study
∆*oxyR*	*oxyR* gene deletion mutant derived from PXO99^A^, Gm^r^	Our lab
∆*oxyR(*pBBR*-oxyR)*	Complementary bacterium strain of ∆*oxyR,* Ap^r^	Our lab
Plasmid		
pMD18-T	Cloning vector, Ap^r^	TaKaRa, Tokyo
pET-28a	Expression vector to generate a N-terminal His_6_ tag, Km^r^	Haigene
pK18mobsacB	Suicidal vector carrying *sacB* gene for mutagenesis, Gm^r^	Schafer et al., 1994 [44]
pBBR1MCS-4	Broad-host range expression vector, Ap^r^	Kovach et al., 1995 [48]
pHM1	Broad-host range expression vector, Sp^r^	Hopkins et al., 1992 [49]
pHT304BZ	Promoterless *lacZ* vector, Ap^r^	Lereclus et al., 1996 [50]
pMDcatB	pMD18-T derivative carrying the full length of *catB*, Ap^r^	This study
pMDcatBr	pMD18-T derivative carrying the right fragment of *catB*, Ap^r^	This study
pMDcatBl	pMD18-T derivative carrying the left fragment of *catB*, Ap^r^	This study
pMDoxyR	pMD18-T derivative carrying the full length of *oxyR*, Ap^r^	This study
pKcatB	pK18mobsacB derivative carrying the full length of *catB*, Gm^r^	This study
pEToxyR	pET-28a derivative carrying the full length of *oxyR*, Km^r^	This study
pBBR-*catB*	pBBR1MCS-4 derivative carrying the full length of *catB*, Ap^r^	This study
pHTpB	pHT304BZ derivative carrying the promoter region of *catB*, Ap^r^	This study
pH-*lacZ*	pHM1 derivative carrying the promoterless *lacZ*, Sp^r^	This study
pH-*catBp-lacZ*	pHM1 derivative carrying the promoter region of *catB* and promoterless *lacZ*, Sp^r^	This study

^a^Ap^r^,Km^r^,Sp^r^,and Gm ^r^indicate resistant to ampicillin, kanamycin, spectinomycin and gentamicin, respectively

### Bioinformatics analysis of CatB

The domain organization of CatB was analyzed using online software available at the SMART Website (http://smart.embl-heidelberg.de/). The amino acid sequences of active CATs, which represent the conserved catalase-domain were obtained from the National Center for Biotechnology Information (NCBI) website. BLASTP was using for searching the homology in *Xanthomonas* species. Relevant sequence alignment was performed using the DNAMAN software (Lynnon Biosoft, San Ramon, USA).

### Expression and purification of OxyR

The full length (942 bp) of *oxyR* (gene ID: PXO_04591) was PCR-amplified using the primer pairs oxyRF/oxyRR (Additional file 5: Table S2). The PCR fragment was gel purified and cloned to the middle vector pMD18-T (Takara, Tokyo, Japan), resulting in construct pMDoxyR, which was verified by DNA sequencing (Beijing Genomics Institute, Beijing, China). The coding region of *oxyR* was digested from pMDoxyR using BamHI and HindIII, and then cloned into expression victor pET28a, resulting in pEToxyR. The recombinant plasmid was transformed into *E. coli* BL21 strains for protein expression. The OxyR purification was performed as previously described [42]. OxyR was induced by addition of isopropyl-thio-galactopyranoside at a final concentration of 0.1 mM and the bacterial culture was then incubated at 20 °C for 6 h. Bacterial cells were chilled at 4 °C and collected by centrifugation. The supernatant containing the soluble protein was collected and mixed with pre-equilibrated Ni2_resin (GE Healthcare, Piscataway, NJ, USA) for 2 h at 4 °C, then placed into a column and extensively washed with buffer containing 1 × PBS and 20 mM imidazole. OxyR was subsequently eluted with buffer containing 100 mM imidazole. The purified OxyR protein was obtained through the gradient dialysis of 1xPBS buffer. The purified OxyR protein was analyzed by sodium dodecyl sulfate polyacrylamide gel electrophoresis.

### Electrophoretic mobility shift assay (EMSA)

The *catB* promoter DNA region (−312 to + 78, the nucleotide site upstream or downstream of translation start (+1)) was amplified by PCR using 5′ ends FAM labeled primers catBpF/catBpR (Additional file 5: Table S2). DNA binding was performed in a 10 μL reaction volume containing EMSA/Gel-Shift Binding Buffer (Beyotime, Shanghai, China), 2 nM labeled DNA fragment and 5 nM His-OxyR protein. Three controls were included in each EMSA experiment: (I) cold probe as specific DNA competitor (unlabeled catB promoter DNA region, 20 nM), (II) negative probe as nonspecific DNA competitor (unlabeled coding region of 16S rRNA gene, 20 nM), and (III) Bovine Serum Albumin (BSA, 5 nM) as nonspecific protein competitor. After incubation at 25 °C for 30 min, the products were loaded onto a native 4 % (W/V) polyacrylamide gel and electrophoresed in 0.5 × TBE buffer for about 1.5 h at 100 V. The fluorescence of samples was detected by Typhoon FLA-5100 (Fuji film, Tokyo, Japan) at 488 nm.

### Construction of *catB* promoter fusion and assay for β-galactosidase

A promoter DNA region (−312 to + 78) of *catB* was obtained by PCR with the PrimeSTAR^®^ Max DNA Polymerase (Takara, Tokyo, Japan) and the primers catBpF and catBpR (Additional file 5: Table S2), while the PXO99^A^ genome DNA as a template. The PCR fragment was cloned directionally into the HindIII and BamHI sites of plasmid pHT304BZ that harbors an ampicillin resistance gene (*Ap* ^r^) and a promoterless *lacZ* reporter gene. The resulting clone pHTpB was verified by DNA sequencing (Beijing Genomics Institute, Beijing, China). Next, pHTpB was treated with HindIII and KpnI, and the fragment containing *catB* promoter region and the promoterless *lacZ* reporter gene was purified with TIANgel Midi Purification Kit (Tiangen, Beijing, China), and then cloned into pHM1, resulting in plasmid pH-*catBp*-*lacZ*. Meanwhile, the fragment of promoterless *lacZ* was obtained from pHT304BZ using BamHI and KpnI and cloned into pHM1, resulting plasmid pH-*lacZ.* These recombinant plasmids were introduced into *Xoo* strians. The plasmid pH-*lacZ* was used as a negative control. *Xoo* strains transformed with the recombinant plasmid were grown in M210 at 28 °C till an optical density (OD_600_) of 1.0 and exposed to 3 mM H_2_O_2_ or sterilized deionized and distilled water (ddH_2_O) for 0.5 h, then harvested by centrifugation at 7,000 g for 5 min. The β-galactosidase activity in the cellular extracts was measured using the β-Galactosidase Enzyme Assay System (Promega, Wisconsin, USA). All assays were performed with three biological replicates and three repeats.

### RNA isolation and quantitative real-time PCR (qRT-PCR) analysis

The transcriptional levels of *catB* and *oxyR* at H_2_O_2_ treatment were detected as described previously with some modifications [42]. Bacterial cells were grown in M210 at 28 °C till an OD_600_ of 0.8 and exposed to 3 mM H_2_O_2_ for 0.5 h, then harvested for analysis of gene expression. Total RNA was extracted with TRIzol reagent (Invitrogen, Carlsbad, CA, USA) and treated with DNase. First-stand cDNA was synthesized from total RNA using the Superscript III reverse transcriptase (Invitrogen, Carlsbad, CA, USA). RT-qPCR was performed using SYBR Green detection reagents (Quanta Biosciences, Carlsbad, CA, USA) in Applied Biosystem’s 7500 Sequence Detection System (Applied Biosystems, Foster City, CA, USA) with the primers (catBqF/catBqR, oxyRqF/oxyRqR), and *gyrB* was used as a reference gene (Additional file 5: Table S2). The relative expression ratio was calculated using 2^–∆∆Ct^ method [43]. All experiments were performed in three biological replicates and triplicate PCR.

### Cloning, deletion and complementation of *catB*

The full length (1,524 bp) of *catB* including ribosome binding site (gene ID: PXO_02830) was amplified by polymerase chain reaction (PCR) using the primer pairs P1/P2 (Additional file 5: Table S2). A right fragment (489 bp) and a left one (582 bp) were amplified by PCR using the primer pairs catBrF/catBrR and catBlF/catBlR (Additional file 5: Table S2), respectively. The PCR fragments were gel purified and cloned to the middle vector pMD18-T (Takara, Tokyo, Japan), resulting in constructs pMDcatB, pMDcatBr, and pMDcatBl, which were verified by DNA sequencing (Beijing Genomics Institute, Beijing, China).

The gene deletion mutant Δ*catB* derived from PXO99^A^ was constructed by the homologous recombination as described previously by using the suicide vector pK18mobSacB [44]. The vector pMDcatBl with the left fragment and the vector pMDcatBr with the right fragment were digested with corresponding restriction enzymes and ligated to pK18-mobsacB. A gentamicin resistance gene (*Gm* ^r^) at 855 bp was then inserted into the intermediate region between left and right fragment carried by pK18mobsacB, resulting in plasmids pKcatB, and then introduced into PXO99^A^ by electroporation. The deletion mutants were screened on PSA plates containing gentamicin and 10 % sucrose. For the complementation experiment, the vector pMDcatB with the *catB* gene including ribosome binding site was digested by enzymes and cloned into pBBR1MCS-4, generating pBBR-*catB*, and then transferred into ∆*catB* by electroporation and screened on PSA plates containing ampicillin.

### Growth curve assay

The bacterial growth assay was performed as previously described [45]. In brief, *Xoo* wildtype, *catB* deletion mutant and complementary strain were grown in M210 liquid medium overnight at 28 °C, then these strains were diluted in M210 medium to a final cell density (OD_600_ = 0.01). The diluted cells were cultured at 28 °C with 200 rpm, and bacterial population was measured after every 6 h. For bacterial population assay, the bacterial cells were spread onto PSA plats after optional diluted, and cultured at 28 °C for 3 days, the bacterial colonies then were counted. These experiments were repeated three times, independently.

### H_2_O_2_ resistance assay

Bacterial strains were cultured in M210 liquid medium using a shaker (200 rpm, 28 °C) until an OD_600_ = 1. For H_2_O_2_ disc diffusion assays, 200 μL of each culture was taken and mixed with 20 mL soft PSA medium containing 0.5 % sodium carboxymethyl cellulose (Sigma-Aldrich, Louis, MQ, USA), then poured out the mixture quickly to PSA medium containing 1 % carboxymethyl cellulose, and ensured the mixture cover the whole PSA plate fully and smoothly. After 5 min, when the mixture became dry, put a sterilized dry Whatman 3MM filter discs of 8 mm diameter on the central of PSA plate. 10 μL of H_2_O_2_ at different concentrations (1, 0.5 and 0.25 M) was spotted onto the discs, respectively. The treated PSA plates were cultured in incubator at 28 °C, and the diameters of H_2_O_2_ inhibition zone were measured after 72 h. For H_2_O_2_ sensitivity assays, 1 mL bacterial cultures were taken and mixed with 100 mL M210 liquid medium, and H_2_O_2_ was then added to the cell suspensions to different concentrations (0, 0.25, 0.5 and 1 mM). The mixtures were cultured at 28 °C on 200 rpm, and the bacterial population was measured at 12 and 24 h, respectively. These experiments were repeated three times with three replicates.

### Catalase activity assay

The analysis of catalase activity was performed as described previously [14, 31]. The bacterial culture conditions were the same as described above. The bacterial concentration of each strain is OD_600_ = 1. The bacterial cells were chilled at 4 °C, collected by concentration at 6,000 g, and then re-suspended in ddH_2_O. Sonication was followed until the bacterial liquid visible clearly. The cell extracts were separated by centrifuge with rate 12,000 g for 30 min, and the most upper layer liquid which contained the protein was transferred to a new tube. 100 μL protein was taken and mixed with 1 mL ddH_2_O, and the optical density of this mixture was measured at 240 nm both before and after adding H_2_O_2_ to the final concentration at 10 mM. The catalase activity was calculated by an extinction coefficient of 43.6 M^−1^ cm^−1^ at 240 nm. One unit of catalase activity was defined as the amount of activity required to decompose 1 μmol of H_2_O_2_ per minute under the assay conditions. The experiments were repeated three times with three replicates.

### H_2_O_2_ detection

The H_2_O_2_ accumulation in *Xoo* strains were detected as previously described. Briefly, *Xoo* strains were grown in M210 liquid medium using a shaker (200 rpm, 28 °C) until an OD_600_ of 1, and harvested by centrifugation at 7,000 g for 5 min. The bacterial cells were re-suspended with 50 mM of potassium phosphate (pH 7.8), and the supernatants were collected by centrifugation again. To measure H_2_O_2_, 0.45 mL of supernatant was mixed with 0.25 mL of 200 μM Amplex red (Sigma-Aldrich, Louis, MQ, USA) and 0.25 mL of 0.02 mg/mL horseradish peroxidase (Sigma-Aldrich, Louis, MQ, USA). The amount of OD_610_ was then measured and converted to H_2_O_2_ concentration using a curve obtained from standard samples. The experiment was repeated three times.

### Histochemical detection of H_2_O_2_ in rice leaves

The cultured bacteria re-suspended in sterilized ddH_2_O at an OD_600_ of 1.0 were prepared as described above, and cells were infiltrated into rice cultivar (*Oryza sativa* L. *subsp. japonica*) leaves grown for two weeks using a needleless syringe. The H_2_O_2_ was detected by DAB staining as previously reported [46]. Briefly, leaf sections (3–5 mm) at 12 and 24 h post-inoculation were cut and placed in water with 0.01 % Triton-X-100 and DAB at 1 mg mL^−1^, then this solution was infiltrated with low vacuum pressure for 10 min and the leaves were incubated for 8 h at room temperature. Finally, leaves were boiled with 95 % ethanol for 10 min and then rinsed with water, and presence of H_2_O_2_ was visualized as reddish brown colored dark spots by a light microscope (Leica, Heidelberg, Germany).

### Pathogenicity test

The bacterial cells were grown in M210 medium for 24 h at 28 °C as described above, and re-suspended in sterilized ddH_2_O at an OD_600_ of 1.0. The susceptible rice cultivar (*Oryza sativa* L. *subsp. japonica*) plants grown for 6 weeks were used for bacterial inoculation with the leaf-clipping method [42]. At least ten leaves were inoculated for each strain, and the experiment was repeated three times. The disease lesion length was recorded 14 d after bacterial inoculation. The top 20 cm of each lesion leaf was cut down and weighted, and then grinded by the sterilized pestle. The extract was gradually diluted by ddH_2_O, and then poured out to PSA medium and cultured in incubator with 28 °C for 3 d after it dry. The bacterial numbers in each plate were accounted, and the bacterial amount in the lesion leaf was calculated.

### Data analysis

All analysis were conducted using SPSS 14.0 (SPSS Inc., Chicago, IL, USA). The hypothesis test of percentages (*t*-test, *P* = 0.05) was used to determine significant differences in the assays for bacterial H_2_O_2_ resistance, catalase activity, endogenous H_2_O_2_ concentration, pathogenicity, gene expression and in vivo growth.

## Additional files

Additional file 1: Table S1. The homologs of *catB* in plant pathogenic Xanthomonas species. (DOCX 15 kb)
Additional file 2: Figure S1. In vitro growth of *Xanthomonas oryzae* pv. *oryzae* wildtype, ∆*catB* and ∆*catB*(*pBBR*-*catB*) strains. The bacterial strains were cultured in M210 liquid medium at 28 °C for 200 rpm, and bacterial population were determined by OD_600_ density measurement at the time points indicated. Data represent the mean and standard deviations of three independent experiments. (TIF 74 kb)
Additional file 3: Figure S2. Coomassie blue staining of recombinant OxyR protein purified from *E. coli* BL21 strains. About 10 μg of protein was loaded in the lane. (TIF 141 kb)
Additional file 4: Figure S3. Histochemical detection of H_2_O_2_ in situ by DAB staining in rice leaves. Rice plants were grown for two weeks and inoculated with ddH_2_O control (A), wildtype (B), ∆*catB* (C), ∆*catB*(pBBR-*catB*) (D), ∆*oxyR* (E), and ∆*oxyR*(pBBR-*oxyR*) (F) by using a needleless syringe. Dark spots represent presence of H_2_O_2_. The experiment repeats three times, independently. (TIF 991 kb)
Additional file 5: Table S2. The information of primers in this study. (DOCX 15 kb)

## Abbreviations

Ap: Ampicillin
*Bs*: *Bacillus subtilis*
BSA: Bovine Serum Albumin
CAT: Catalase
CFU: Colony forming units
DAB: 3, 3′-diaminobenzidine
EMSA: Electrophoretic mobility shift assay
Gm: Gentamycin
H_2_O_2_: Hydrogen peroxide
Km: Kanamycin
NCBI: National Center for Biotechnology Information
OD: Optical density
PCR: Polymerase chain reaction
PSA: Peptone sucrose agar
qRT-RCR: Quantitative real-time PCR
ROS: Reactive oxygen species
Sp: Spectinomycin
U: Unit
*Xac*: *Xanthomonas axonopodis* pv. *citri*
*Xoo*: *Xanthomonas oryzae* pv. *oryzae*
