# Supplementary material for: OxyR-regulated catalase CatB promotes the virulence in rice via detoxifying hydrogen peroxide in Xanthomonas oryzae pv. oryzae
Source: BMC Microbiol. 2016 Nov 8;16:269. doi: 10.1186/s12866-016-0887-0 (PMC5101826; doi:10.1186/s12866-016-0887-0)
Supplement: Additional file 1: Table S1. — The homologs of catB in plant pathogenic Xanthomonas species. (DOCX 15 kb) [file 12866_2016_887_MOESM1_ESM.docx]

**Table S1 Homologues of *catB* in several plant pathogenic Xanthomonas species**

| **Bacterial strains** | **Homologue** | | | **Identify (％)** |
| --- | --- | --- | --- | --- |
|  | **Gene/locus_tag** | **Putative product** | **Size (aa)** |  |
| *Xanthomonas oryzae* pv. *oryzae* MAFF311018 | XOOD379 | Catalase | 507 | 99.8 |
| KACC10331 | AAW73671 | Catalase | 507 | 99.8 |
| PXO86 | AZ54_23040 | Uncharacterized protein | 507 | 99.8 |
| *X*. *campestris* pv. *campestris* | KatB | Catalase | 507 | 96.4 |
| *X*. *campestris* pv. *vesicatoria* 85-10 | CatB | Catalase | 507 | 97.0 |
| *X*. *campestris* pv. *phaseoli* | PK68_04415 | Catalase | 507 | 97.0 |
| *X*. *perforans*  91-118 | XPE_3530 | Catalase | 492 | 97.0 |
| *X*. *fuscans* subsp. *aurantifolii*  ICPB 10535 | CatB | Catalase | 507 | 96.6 |
| *X*. *arboricola* pv. *pruni* | DK27_14900 | Catalase | 501 | 95.8 |
| *X*. *hortorum* pv. *carotae*  M081 | XHC_4112 | Catalase | 507 | 95.7 |
| *X*. *fragariae*  LMG25863 | O1K_10842 | Catalase | 507 | 93.1 |
