# Supplementary figures and images for: OxyR-regulated catalase CatB promotes the virulence in rice via detoxifying hydrogen peroxide in Xanthomonas oryzae pv. oryzae
Source: BMC Microbiol. 2016 Nov 8;16:269. doi: 10.1186/s12866-016-0887-0 (PMC5101826; doi:10.1186/s12866-016-0887-0)

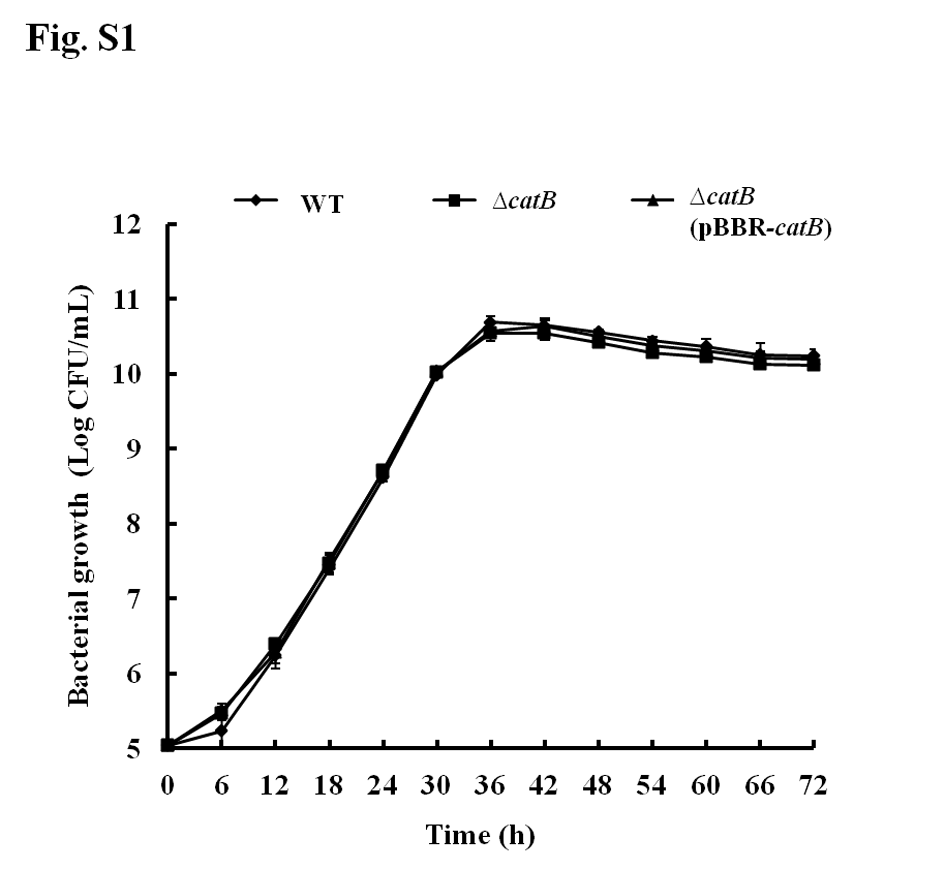

Supplement: Additional file 2: Figure S1. — In vitro growth of Xanthomonas oryzae pv. oryzae wildtype, ∆catB and ∆catB(pBBR-catB) strains. The bacterial strains were cultured in M210 liquid medium at 28 °C for 200 rpm, and bacterial population were determined by OD600 density measurement at the time points indicated. Data represent the mean and standard deviations of three independent experiments. (TIF 74 kb) [file 12866_2016_887_MOESM2_ESM.tif]

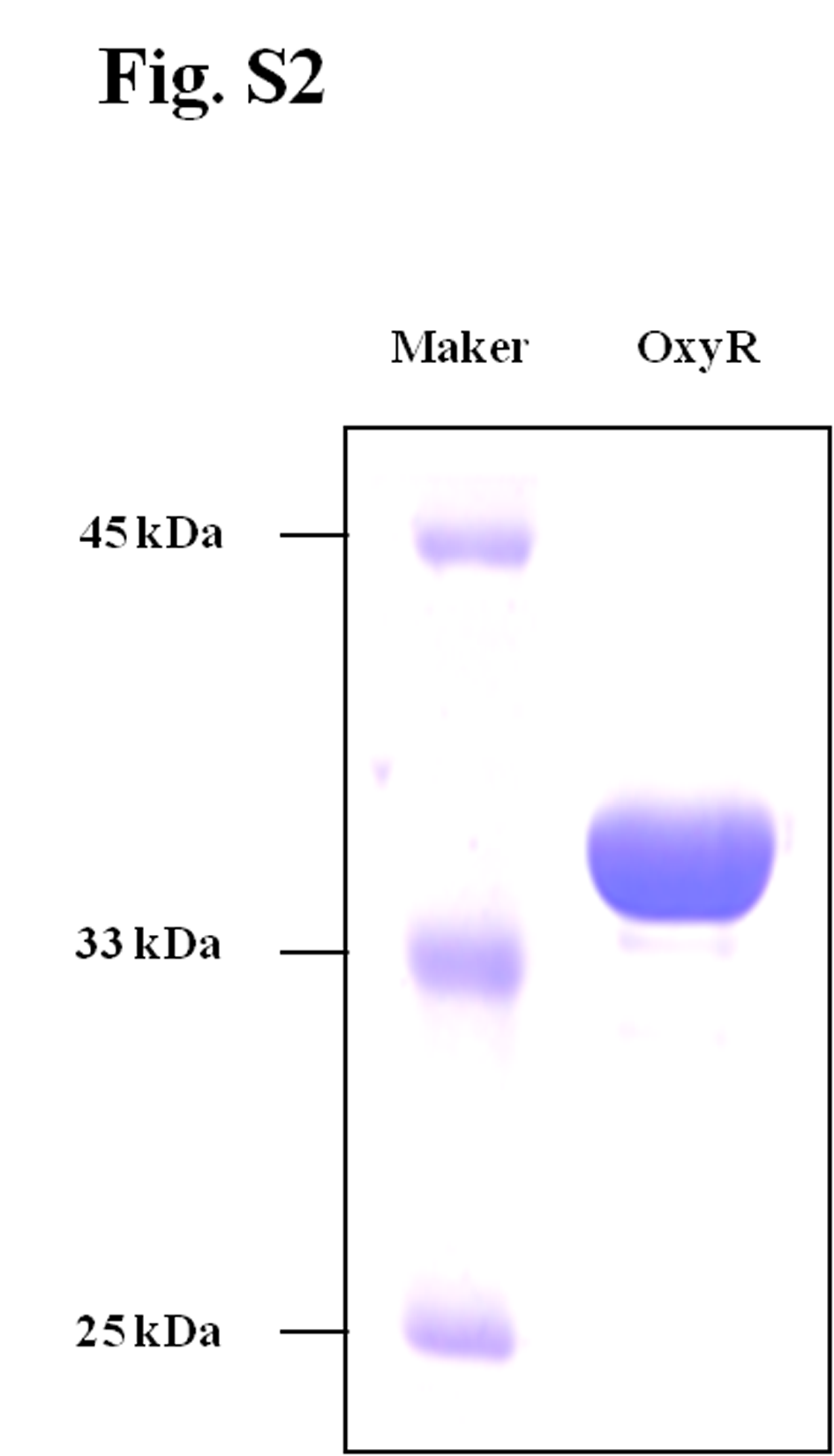

Supplement: Additional file 3: Figure S2. — Coomassie blue staining of recombinant OxyR protein purified from E. coli BL21 strains. About 10 μg of protein was loaded in the lane. (TIF 141 kb) [file 12866_2016_887_MOESM3_ESM.tif]

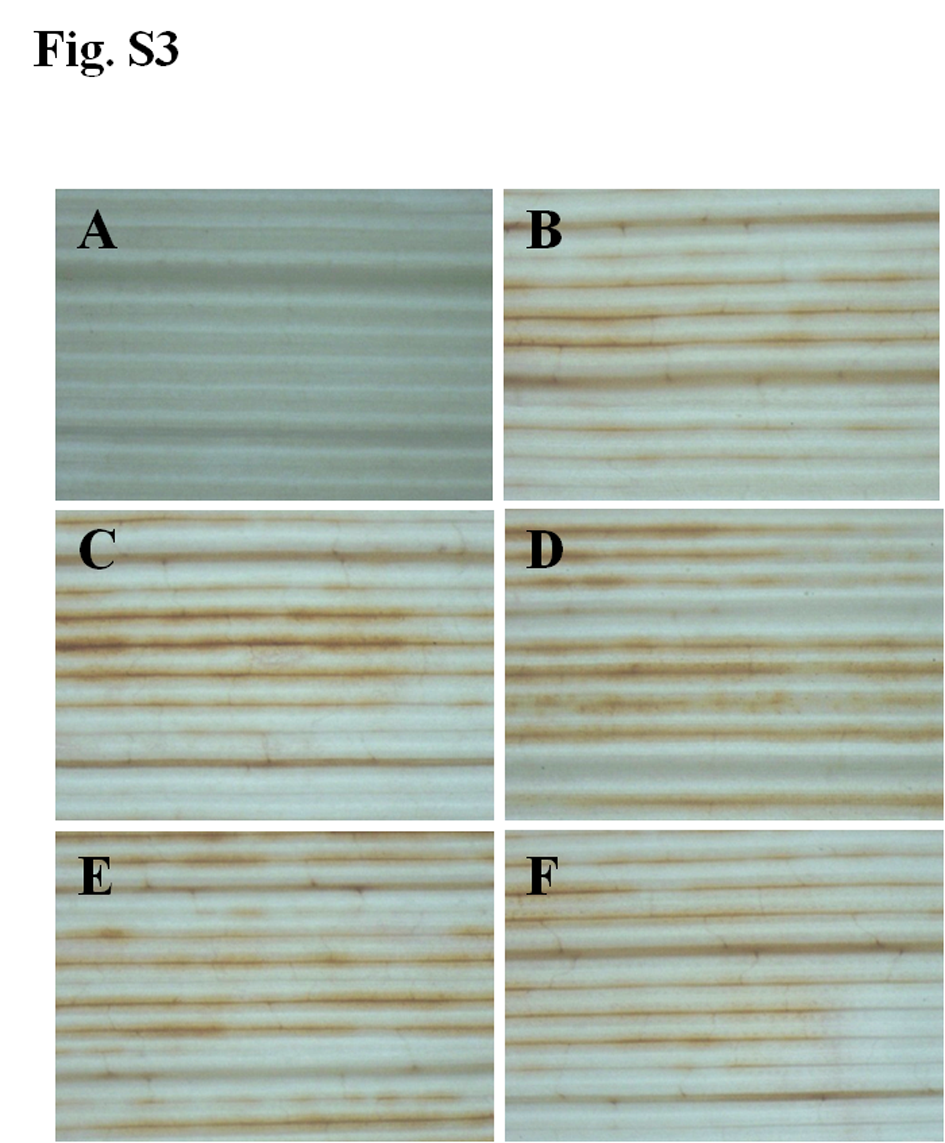

Supplement: Additional file 4: Figure S3. — Histochemical detection of H2O2 in situ by DAB staining in rice leaves. Rice plants were grown for two weeks and inoculated with ddH2O control (A), wildtype (B), ∆catB (C), ∆catB(pBBR-catB) (D), ∆oxyR (E), and ∆oxyR(pBBR-oxyR) (F) by using a needleless syringe. Dark spots represent presence of H2O2. The experiment repeats three times, independently. (TIF 991 kb) [file 12866_2016_887_MOESM4_ESM.tif]
