# Supplementary material for: OxyR-regulated catalase CatB promotes the virulence in rice via detoxifying hydrogen peroxide in Xanthomonas oryzae pv. oryzae
Source: BMC Microbiol. 2016 Nov 8;16:269. doi: 10.1186/s12866-016-0887-0 (PMC5101826; doi:10.1186/s12866-016-0887-0)
Supplement: Additional file 5: Table S2. — The information of primers in this study. (DOCX 15 kb) [file 12866_2016_887_MOESM5_ESM.docx]

**Table S2. The primers in this study**

| **Primer** | **Sequences( 5′- 3′ )** | **Restriction enzyme/use** |
| --- | --- | --- |
| P1 | TCGAGCTCTGTTCATTGATCGTAACC | SacI / Gene cloning |
| P2 | CCAAGCTTTCAGTCCTGCAGGCTGGA | HindIII / Gene cloning |
| catBlF | CGGGATCCGGCGTCAGCTCGTTTGAG | BamHI / Gene deletion |
| catBlR | CGGAATTCTGGTTTGAACGGTGGG | EcoRI / Gene deletion |
| catBrF | CCAAGCTTTTGCCCTGCCCGTCCTT | HindIII / Gene deletion |
| catBrR | GCTCTAGATCCGCCCGACGACATCGC | XbaI / Gene deletion |
| oxyRF | CGGGATCCATGAATCTGCGTGACCTGAAATATC | BamHI / |
| oxyRR | CCAAGCTTCTAAGCCGCAACCGCCTTC | HindIII / |
| catBpF | TCAAGCTTATCACCGAGCGGGCACCT | HindIII / gene fusion |
| catBpR | TCGGATCCAACGGATTGGGCGAAGAC | BamHI / gene fusion |
| catBqF | ATCGCTTCATGGATGGCAATGG | - / qRT-PCR |
| catBqR | TCGTTGGTCAGGTGGCTGTAG | - / qRT-PCR |
| oxyRqF | ATCCGCCTGATCCGCTTCC | - / qRT-PCR |
| oxyRqR | CTAAGCCGCAACCGCCTTC | - / qRT-PCR |
| gyrBqF | GCGAGCACAATGGCATT | - / qRT-PCR |
| gyrBqR | CCATCCTTCTGCGGGATGT | - / qRT-PCR |
